# Analysis of the available funds supporting marine activities in some key European Mediterranean countries

**DOI:** 10.3389/frma.2022.927383

**Published:** 2022-11-04

**Authors:** Monica Gambino, Paolo Accadia, Marco Costantini, Marina Gomei, Loretta Malvarosa, Evelina Carmen Sabatella, Rosaria Felicita Sabatella

**Affiliations:** ^1^NISEA Research Cooperative, Salerno, Italy; ^2^WWF Mediterranean, Rome, Italy

**Keywords:** subsidies, overcapacity, overfishing, small scale fishery, fishery policy, large scale fishery

## Abstract

The study presented in this article analyzed qualitative and quantitative data on the performance of the European Maritime and Fisheries Fund (EMFF) based on the information reported in the European Union (EU) List of Operations updated to December 2020. Each EMFF measure and type of financial support were divided into three broad categories of subsidies according to their main objectives and scope: capacity enhancing, beneficial, or ambiguous. Capacity enhancing is defined as funds that could incentive overcapacity or overfishing. Beneficial refers to subsidies that have a positive impact on fish stocks and the environment. Ambiguous subsidies correspond to funds that may lead to positive or negative impacts on the environment depending on how they are designed and implemented. The assessment revealed the asymmetric distribution of EMFF resources in the Mediterranean region. In the six member states investigated, EMFF support is concentrated on a limited number of more easily accessible measures from an administrative and financial point of view. Most of the allocated funds are classified as capacity enhancing; other frequently used measures are in the ambiguous category. Small-scale vessels using static gear and accounting for the largest part of the Mediterranean fleets received a negligible share of specific funds for promoting environmentally sustainable fisheries. Most investments are concentrated on larger trawlers to support the temporary cessation of fishing activities and scrapping operations. Further qualitative analysis based on the findings and recommendations of previous reports evaluating the use of EMFF as well as interviews with beneficiaries highlighted that complex administrative procedures and legal uncertainty in the interpretations of some articles of the EMFF regulations are the main reasons for the asymmetric performance of the EMFF measures. The dispersion of responsibilities among European, national and regional authorities, and an evident lack of coordination among them are the main shortcomings that were identified. The limited use of advance payments, the lack of capacity, and technical assistance and obstacles to accessing financial instruments have penalized most of the projects that are focused on innovation, diversification, and environmental sustainability.

## Introduction

This study investigated the allocation of funding from the European Maritime and Fisheries Fund (EMFF) related to the European Union (EU) maritime and fisheries policies for the fishery sector in the Mediterranean region from 2014 to 2020 (Regulation (EU) No 508/2014, 2014). The analysis focused on six Mediterranean Member States (MSs) (Italy, Croatia, Greece, Malta, Spain, and France)^1^ which, at the end of 2018, overall accounted for 99% of the EU landings (in weight and value) and 98% of the EU fleet operating in the Mediterranean region (https://datacollection.jrc.ec.europa.eu).

The EMFF was endowed with a total budget of €8.6 billion, 74% of which is contributed by the EU (€6.4 billion). The remaining €2.2 billion is contributed by the MSs (https://cohesiondata.ec.europa.eu/funds/emff). The allocation of funds is subdivided into six Union Priorities (UPs) over a 7-year period (2014 to 2020). The fishery sector mainly falls under the UP 1 category: promoting environmentally sustainable, resource-efficient, innovative, competitive, and knowledge-based fisheries. That category is allocated €2.4 billion, which is the largest share (28%) of the total EMFF budget. The MSs, which represent 89% of the EU's contribution to the EMFF, have submitted an operational programme to the Commission establishing how the funds would be used during the programming period (EU Commission Implementing Decision 2014/372/EU, 2014).

Each year, countries are required to publish relevant information on their websites, including information on the beneficiaries of the funds as per Article 119 of Regulation (EU) No 508/2014 (2014). The assessment of the fishery subsidies in the Mediterranean regions reported in this study is based on the EMFF List of Operations updated to 2020.

The subsidy categories were based on the criteria provided by Sumaila et al. (2019a) and Skerritt et al. (2020). Each EMFF measure and type of financial support were divided into three broad types of subsidies according to their expected impact and scope of intervention: capacity enhancing, beneficial, or ambiguous. Capacity enhancing is defined as funds that could incentive overcapacity or overfishing, artificially increasing the profitability of recipient fleets (Sumaila et al., 2019b). Beneficial refers to subsidies that have a positive impact on fish stocks and the environment. Ambiguous subsidies are funds that may lead to positive or negative impacts on the health of fish stock or the environment depending on how they are designed and implemented. The EU Fisheries and Aquaculture Monitoring and Evaluation (FAME) toolkit created for the ex-post evaluation of EMFF was used as a reference for the complementary classification of EMFF measures in terms of their impact on the environment (EU, 2017a).

The analysis on the use of public fishery funds in the Mediterranean region was conducted using four methods. First, a quantitative assessment of fishery support measures to MSs in the Mediterranean region in 2014–2020 was performed by evaluating the performance and results by fleet segment and some specific programmes. Then, an in-depth analysis of the efficiency of the fishery subsidies was conducted based on additional economic and biological indicators available at the fleet segment level and using the result indicators suggested by the FAME guidelines for the ex-post evaluation of EMFF (EC, 2017). This analysis aimed to delve deeper into the details used to draw conclusions about the efficacy of EMFF funds in 2014–2020. Next, intensity metrics were used to further evaluate the level of subsidization for the six countries covered by the analysis. Finally, the results were complemented by qualitative considerations of the EU budgeting measures based on four case studies representing examples of successful projects in relation to the protection of the marine environment. The interviews provided supplementary information about best practices as well as critical feedback on the implementation of some of the EMFF measures (Supplementary material).

## Materials and methods

### Data collection

Data and information available on subsidies from 2014 to 2020 were collected from the EMFF List of Operations for Croatia^2^, France^3^, Greece^4^, Italy^5^, Malta^6^, and Spain^7^. The EMFF List of Operations (EU Commission Implementing Decision, 2014/464, 2014) provides data on eligible expenditures and EU contributions in each MS, including details on the beneficiaries and the typology of the measure. The following information is included (Table 1): name of the recipient of the funds, community fleet register identification number (if the project is linked to a fishing vessel), name of the project, brief summary describing the project, start and expected completion date for the project, total amount of the eligible costs, total size of the funds awarded and the source of the funding, postcode, and country in which the project is based and the UP related to the project.

**Table 1:** Bridging table between the list of operations and the variables included in the aggregated database.

**Data field from list of operation**	**Variables in the data base**
Operation postcode	NUTS 2
Name of Union priority, operation name operation summary	ID Measure
Beneficiary name, Community fleet register	Beneficiaries' categories (Operators, Public bodies, Other)
Total eligible expenditure (EUR)	Decided: Total financial resources allocated to selected projects (project pipeline)
Amount of Union contribution (EUR)	The union contribution of total eligible expenditure /EU total allocation)
Payments (EUR)	Total financial execution

All the available information gathered from official sources was processed to build a database as listed below:

- The EU Nomenclature of Territorial Units for Statistics (NUTS), required to distinguish the Spanish and French Mediterranean regions, were obtained from the postal code of the operations' municipalities.
- The type of the EMFF measure was taken from references to the name of the UP and details on the operations (name & summary).
- The beneficiaries' category was obtained from the beneficiary name and community fleet register fields.
- The total financial resources allocated to selected projects (decided amounts) correspond to the total eligible expenditure.
- The contribution of the total eligible expenditure corresponds to the EU's total allocation.
- Where available (such as for France, Italy, and Greece), the financial execution field was taken or derived from the information on payments.

The expenditure amounts are presented in current values because the 2020 inflation rates were not available when the analysis was performed. Nonetheless, considering the stability of prices over the studied period (2014–2020), this assumption should not have significatively impacted the results of the analysis.

The collected data were complemented with stakeholder interviews to provide additional information and perspectives on the EMFF programme. Thus, the director of a Marine Protected Area (MPA) for the Southern Tyrrhenian coast, two researchers involved in a project for a cuttlefish fishery in the Northern Adriatic Sea, and the managers of an Italian and a Spanish Fishery Local Action Group (FLAG) were interviewed.

### Methodology for the allocation of EMFF funding by fishing vessels

Information on EMFF measures was cross-checked with the list of vessels included in the Union Fishing Fleet Register on 31 December 2020 (https://webgate.ec.europa.eu/fleet-europa/search_en), based on the Community Fleet Register (CFR) number, which is the unique identification number of a fishing vessel, and the link between information provided in the national operations lists and those included in the CFR with specific reference to the main gears and the length overall (LOA) in meters of the community fishing vessels^8^.

The criterion for allocating a vessel in large-scale fleet (LSF) segments carrying out trawling and small-scale fleet segments is based on the field concerning the main fishing gear in the fleet register: if the main fishing gear reports that a net is used for trawling^9^ (e.g., bottom trawl, midwater pair trawl, and beam trawl), it was assumed that the vessels are trawlers. Analogously, vessels with an LOA ≤ 12 meters are classified in the length class <=12 meters. It is important to emphasize that, whereas the classification according to the LOA is univocal, as it is based on the physical characteristics of the vessels, this is not the case of the criterion based on the main fishing gear, which corresponds to the information indicated in the fishing license at the time the vessel was enrolled in the register. However, the allocation of vessels into fleet segments within the EU's data collection framework (DCF) is based on the predominance in the actual use of fishing gear during the year^10^, which might vary over the years in polyvalent contexts where fishing vessels use different gears according to the fishing seasons. Therefore, the gear classified as main in the register could be not updated and not perfectly reflect the current segmentation of the fleets analyzed. Consequently, the classification of vessels based on the main fishing gear as provided by the EU fleet register was further cross-checked with the fleet segmentation reported in the Annual Economic Report (STECF, 20-11). This also allows for a comparison of the distribution of the fleet based on the main fishing activity (small-scale coastal fleet [SSCF] and LSF, according to EU DCF^10^).

It is worth noting that, for some of the EMFF measures designated to fishing vessels, information taken from the operations lists was misreported or did not include the CFR number, as was the case for Greece.

### Classification of the subsidies

According to the criteria provided by Sumaila et al. (2019a) and Skerritt et al. (2020), the EMFF measures were reclassified as beneficial, capacity enhancing, and ambiguous. Beneficial subsidies are the programmes included in UP1—promoting environmentally sustainable, resource, efficient, innovative, competitive, and knowledge-based fisheries—except for subsidies for the cessation or interruption of fishing activities and forms of capital inputs and infrastructure investments. Capacity-enhancing subsidies include all programmes belonging to UP5: funds that facilitate marketing and processing and those with the potential to encourage fishing capacity as the measure related to energy efficiency (EMFF Art. 41) or the construction of ports and landing facilities (EMFF Art. 43). Ambiguous subsidies have the potential to lead to either sustainable management or overexploitation of the fishery resource, depending on how these programmes are delivered. This category includes temporary and permanent cessation of fishing activities (EMFF Art. 33 and Art. 34) and all programmes belonging to UP4: increasing employment and territorial cohesion.

Table 2 presents information on the impact each strategic EMFF measure has on the environment according to a specific field. The measures were associated with the categories based on the impact they can have on the environment and/or on other aspects (e.g., business development, human capital knowledge, institutional capacity building, innovation, and Community Local Led Development [CLLD]). The impact is classified based on whether the effect is direct (primary) or indirect (secondary). The FAME toolkit, for the ex-post evaluation of EMFF, was used as a reference (EC, 2017). Table 2 reports the EMFF measures envisaged to have a direct or secondary impact on the environment and classified according to the categories proposed by Sumaila et al. (2019a).

**Table 2:** EMFF measures that may have an impact on the environment based on the subsidy category.

**Subsidies category**	**EMFF measures**	**Impact on environment**
Beneficial	36. Ensuring of a balance between fishing capacity and available fishing opportunities: Support for the systems of allocation of fishing opportunities	2
	37. Support for the design and implementation of conservation measures and regional cooperation	1
	38. Limitation of the impact of fishing on the marine environment and adaptation of fishing to the protection of species	1
	39. Innovation linked to the conservation of marine biological resources	2
	40. Protection and restoration of marine biodiversity and ecosystems and compensation regimes in the framework of sustainable fishing activities	1
	76. Control and enforcement	2
	77. Data collection	2
	80. Fostering the “Integrated Maritime Policy”	2
Ambiguous	33. Temporary cessation of fishing activities	2
	34. Ensuring of a balance between fishing capacity and available fishing opportunities: Permanent cessation of fishing activities	1
Capacity enhancing	41. Energy efficiency and mitigation of climate change	1
	43. Fishing ports, landing sites, auction halls and shelters	1

Elaboration on (EC, 2017) and on the study's dataset.

It is interesting to observe that measure 34 (permanent cessation of fishing activities), which is supposed to have a direct (positive) impact on the environment (by reducing overcapacity), is classified by Sumaila et al. (2019a) as ambiguous. More importantly, the measure ex-post Art. 41 on energy efficiency, which is supposed to contribute to the mitigation of climate change by subsidizing energy efficiency projects, is classified as capacity enhancing.

### Semi-structured qualitative interviews

To collect additional information and perspectives on the EU subsidy policy, the quantitative assessment of the EMFF was complemented by semi-structured qualitative interviews with relevant actors: six stakeholders who have received beneficial subsidies and have an interest in the environmental sustainability and fishery sector.

The interviews followed a common structure and were organized according to the following guide:

1. Beneficiaries' profile/respondent's position in the organization.
2. Type and number of measures/operations financed under EMFF.
3. Experience with the previous programming period.
4. Main difficulties encountered when applying for EMFF funds.
5. Main risks of losing the funding.
6. Main benefits received by the operations funded.
7. Suggestions for adjustments for the next programming period.

Given the difficulty of accessing stakeholders during the coronavirus (COVID-19) pandemic, phone interviews were conducted; each interview lasted between 45 and 60 min. An interview guide with seven main points was developed; it only required a few iterative changes, as is typical for semi-structured interviews (Berry, 1999).

Although limited to four case studies (three in Italy and one in Spain), the interviews sought to gauge the stakeholders' experiences with European funding and to investigate their respective needs and their expectations of the next programming period.

## Results

### Quantitative assessment of the fund allocations based on EMFF measures

**Figure 1:**
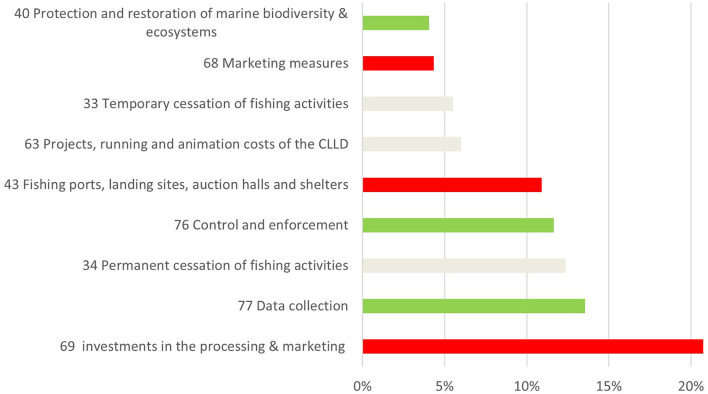
Top 10 EMMF measures in six Mediterranean countries based on percentage of total financial allocation. Elaboration on data from the operations lists of Croatia, Mediterranean France, Greece, Italy, Malta, and Mediterranean Spain until December 2020 (red bar = capacity enhancing, green bar = beneficial; gray bar = ambiguous).

The quantitative data analysis conducted on the operations lists of Italy, Croatia, Malta, Greece, Mediterranean France, and Mediterranean Spain showed that, although EMFF support is distributed among 27 measures, the top 10 measures, in terms of allocated funds, represent more than 90% (€1,250 million) of the total financial budget allocated in the six countries for 2014–2020 (Figure 1).

Capacity-enhancing subsidies, absorbing 38% of the total allocated funds, are mainly represented by the investments in processing (EMFF Art. 69), measures supporting the market dimension of the fisheries policy (EMFF Art. 68), and investments improving the infrastructure of fishing ports, auctions halls, landing sites, and shelters (EMFF Art. 43). One-quarter of the fishery funds are allocated to ambiguous category; of these scrapping operations (EMFF Art. 34), temporary cessation operations (EMFF Art. 33), and projects to implement CLLD strategies (EMFF Art. 63) received most of the funding.

For the beneficial category, EMFF funds are concentrated on projects that comply with the CFP rules regarding data collection (EMFF Art. 77) and control and enforcement (EMFF 76). Only four EMFF beneficial measures (Art. 37- Support for the design and implementation of conservation measures and regional cooperation, Art. 38-Limitation of the impact of fishing on the marine environment and adaptation of fishing to the protection of species, Art. 40 - Protection and restoration of marine biodiversity and ecosystems and compensation regimes in the framework of sustainable fishing activities and Art. 80-Fostering the Integrated Maritime Policy) out of 16 subsidies are directly linked to the marine environment in terms of field of intervention according to FAME criteria for the ex-post evaluation of EMFF (EU, 2017b). Beneficial environmental subsidies account for 5% of the total EMFF funds allocated in the six countries that were surveyed.

**Figure 2:**
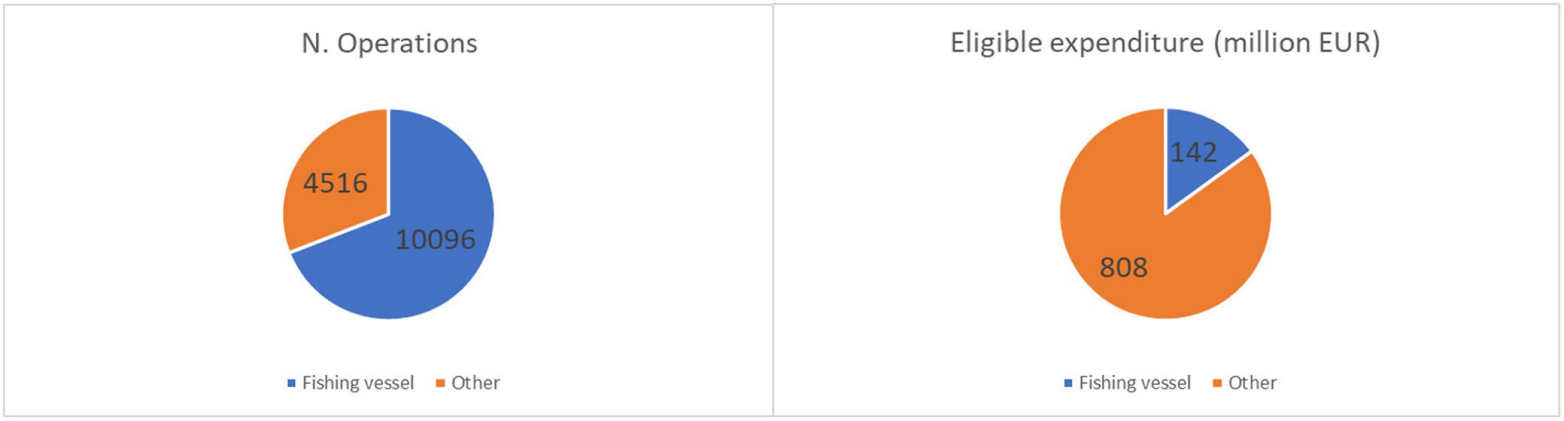
EMFF measures related to the number of operations and eligible expenditure based on the typology of the beneficiary. Elaboration on data from the EMFF List of Operations and the EU Fleet Register of Italy, Croatia, Malta, Mediterranean Spain, and Mediterranean France.

Focusing on the UP1 measures, which are specifically designated to commercial fishing vessels, EMFF information highlighted that direct support for the fishing vessels of the five countries represents 69% of the total financed operations (14,612 operations) and 15% of the total eligible expenditure (€951 million), as shown in Figure 2. Greece was excluded as it was not possible to identify the beneficiary vessels because no field in the database was suitable as a cross-referencing criterion.

**Figure 3:**
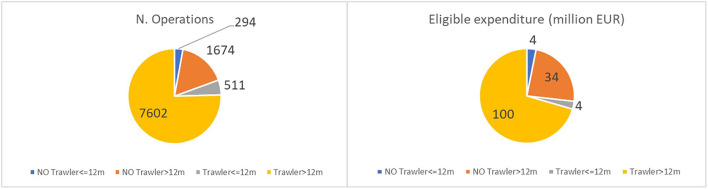
EMMF measures related to the number of operations and eligible expenditure based on main fishing gear and length class. Elaboration on data from the EMFF List of Operations and the EU Fleet Register of Italy, Croatia, Malta, Mediterranean Spain, and Mediterranean France.

The category of trawlers longer than 12 m, accounting for 14% of all registered fleets in the five countries considered, received 75% (7,602 operations) of the total approved projects and 71% (€100 million) of the total budget granted to fishing vessels. Non-trawling vessels longer than 12 m accounted for 17% of the total number of operation and 24% of the eligible expenditure allocated to fishing vessels. Vessels using static fishing gear with a length ≤ 12 m, which are classified as an SSCF and which represent three-quarters of all the registered fleets in the five Mediterranean countries, received 3% of the total EMFF funding specifically designated for vessels in terms of the number of operations and the allocated amount (Figure 3). Although this total is affected by the fact that most small vessels are part of cooperatives and may have received funding through their organization, there is a clear imbalance in the use of public funding between small-scale vessels and large-scale vessels.

**Figure 4:**
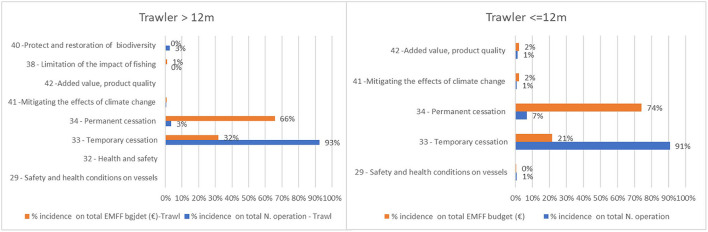
EMMF measures allocated to the trawl fleet. Elaboration on data from the EMFF List of Operations and the EU Fleet Register of Italy, Croatia, Malta, Mediterranean Spain and Mediterranean France.

European Maritime and Fisheries Fund resources for large and small trawlers almost entirely depend on the temporary cessation of fishing activities (Art. 33) and for permanent cessation (Art. 34) (Figure 4). The permanent cessation of activity is the measure with the highest cost per operation due to the premiums paid for it. Financial compensations for the temporary cessation in 2020 also supported the stoppage of all fishing activities caused by the COVID-19 outbreak^11^.

**Figure 5:**
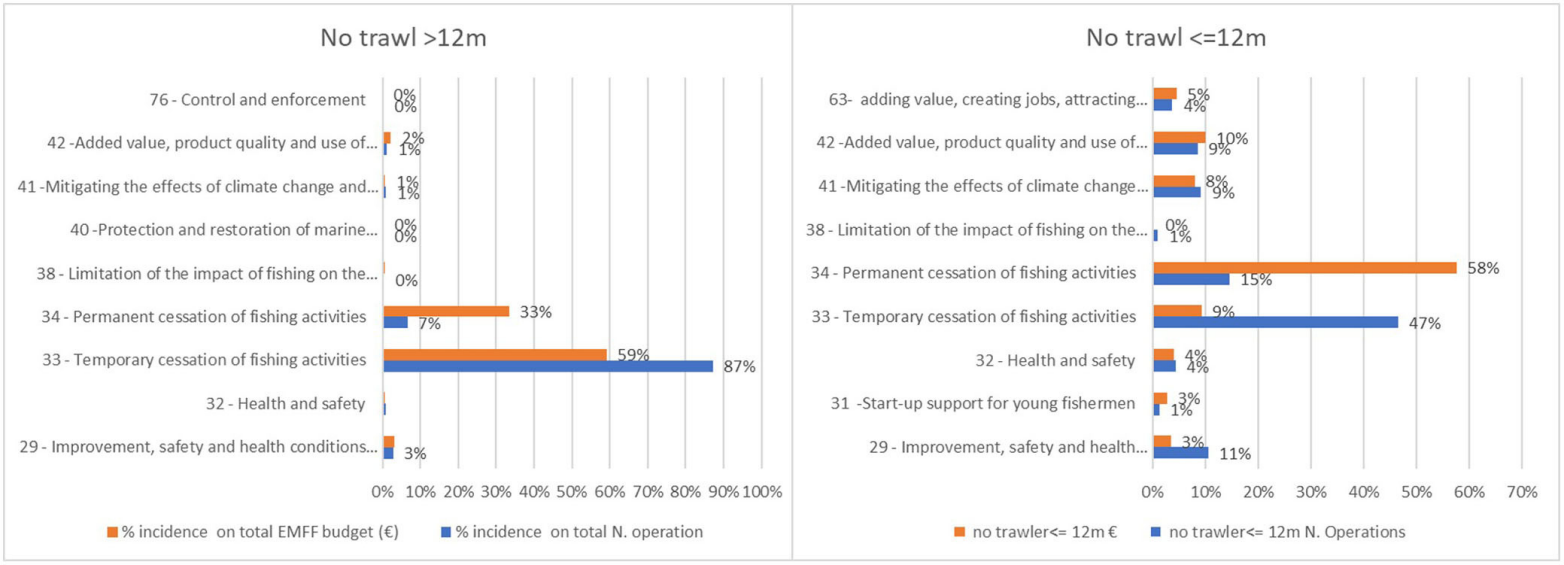
EMMF measures allocated to vessels not equipped with trawl nets. Elaboration on data from the EMFF List of Operations and the EU Fleet Register of Italy, Croatia, Malta, Mediterranean Spain, and Mediterranean France.

The distribution of the EMFF measures to vessels not using trawl nets, especially for smaller vessels, is more balanced and is characterized by greater access to projects aiming to improve the quality of the fishery products (Art. 42), safety and health onboard the vessel (Art. 32), implementation of CLLD strategies (Art. 63), energy efficiency and mitigation of climate change (Art. 41), and promotion of human capital, job creation, and social dialog (Art. 29) (Figure 5).

### Deeper analysis on the impact of EMFF measures under Art. 34 and Art. 41

According to the criteria provided by Skerritt et al. (2020), ambiguous subsidies have the potential to lead to either sustainable management or overexploitation of the fishery resource, depending on how these programmes are delivered. If permanent cessation of fishing activities occurs due to the scrapping of vessels, the evaluation of this measure must be assessed using biological, economic, and technical indicators differentiated by fleet segmentation of the fishing vessels to verify if the objective of achieving a balance between the fleet capacity and the fishing opportunities has been fulfilled (STECF, 20-10).

Based on the same criteria, capacity-enhancing subsidies include all programmes having the potential to encourage fishing capacity regardless of whether they have a positive impact on the environment by reducing energy use and waste. Measures financing engine replacement under Art. 41.2, falling under the objective of energy efficiency and mitigation of climate change, belong to this typology.

To further analyse the efficacy of the measures mentioned above and to understand whether they are ambiguous and/or harmful (capacity enhancing), they were evaluated against the indicators identified by the FAME guidelines for the ex-post evaluation (EU, 2017b).

The analysis presented in this section used the dataset elaborated in the study, but it was limited to the Italian beneficiaries of EMFF funds because the authors only had access to information on the Italian fleet related to the fleet segment category for each vessel included in the list of beneficiaries. In fact, this type of analysis needs details about the beneficiary boats to compare the expected benefits of the funds with the trend of key performance indicators. Specifically, for the ex-post evaluation of projects financed under Art. 34 and Art. 41, the FAME guidelines foresee the following indicators:

- Change in the % of the unbalanced fleet for the permanent cessation of fishing activities (Art. 34).
- Impact of the change in the fuel efficiency of the fish capture on energy efficiency (Art. 41).

#### Permanent cessation

A total of 201 Italian fishery operations were funded under Art. 34 for an overall amount of more than €55 million. As expected, considering that overfishing of the demersal species is prevalent (STECF, 20-10), a higher percentage of funds has been allocated to trawl fleets (demersal trawlers and beam trawlers). Specifically, 82% of the beneficiaries (165) are demersal trawlers (receiving 80% of the total funds allocated to permanent cessation from 2014 to 2020) (Table 3).

**Table 3:** Number of operations and allocated funds under EMFF Art. 34 by Italian fleet segment for the 2014–2020 period.

**Fleets**	**No. operations**	**% on No. operations**	**Allocated funds (€)**	**% on allocated funds**
Dredges (DRB)	2	1%	216.830,00	0%
Demersal trawl (DTS)	165	82%	44.454.770,00	80%
Polivalent passive gears (PGP)	5	2%	366.650,00	1%
Purse seines (PS)	8	4%	2.648.580,00	5%
Beam trawlers (TBB)	6	3%	1.877.970,00	3%
Pelagic trawlers ™	15	7%	5.987.870,00	11%
Total	201,00	100%	55.552.670,00	100%

Elaboration on data from the EMFF List of Operations and the EU Fleet Register as of December 2020 for Italy.

With around €45 million, EMFF has funded the permanent cessation of 7% of the overall demersal trawl fleet (165 out of 2,264 vessels) operating at the beginning of the financing period (Table 4). Most (88%) of the financed operations refer to beneficiary boats falling under the length classes of 12–18 and 18–24 meters, totalling 82% of the overall allocated funds under Art 34 for demersal trawlers. Considering that the permanent cessation financing is proportional to the vessel dimension, as expected, the highest percentage (53%) of funds was allocated to vessels in the length class of 18–24 meters.

**Table 4:** Number of vessels in 2014, number of operations and allocated funds under EMFF Art. 34 to the Italian trawl fleet.

**Fleet segments**	**No. of vessels, 2014**	**% no. Vessels**	**No. Funded operations**	**% on No. operations**	**Allocated funds (€)**	**% on allocated funds**
DTSVL0612	183	8%	6	4%	527.290,00	1%
DTSVL1218	1.254	55%	76	46%	12.774.310,00	29%
DTSVL1824	632	28%	70	42%	23.744.050,00	53%
DTSVL2440	195	9%	13	8%	7.409.120,00	17%
Total	2.264,00	100%	165	100%	44.454.770,00	100%

Elaboration on data from the EMFF List of Operations and the EU Fleet Register as of December 2020 for Italy and Annual Economic Report (STECF, 20-06).

One result of the permanent cessation measure can be observed in the trend of the number of vessels using a demersal trawl as their predominant gear; overall, this decreased by 11% during the 2014 −2019 period (around 250 units less in 2019 than in 2014).

**Figure 6:**
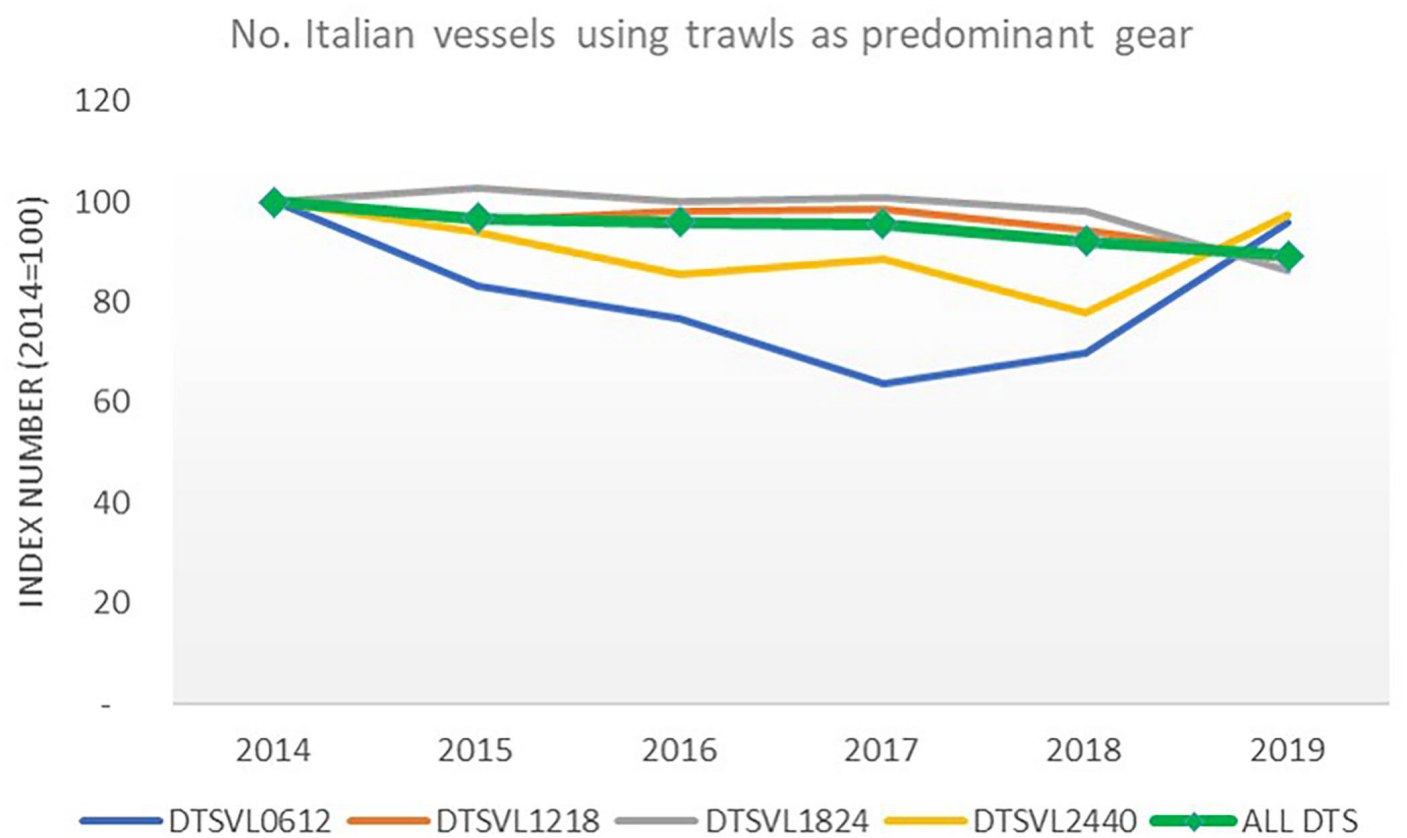
Number of vessels using trawl nets as their predominant gear from 2014 to 2019 in Italy. Elaboration on data from STECF (2020-06) and STECF (20-10).

The highest decrease in rates was observed for demersal trawlers in the length class of 12–18 meters (DTS 12–18) and trawlers in the length class of 18–24 meters (DTS 18–24). These two segments received the highest share of subsidization for permanent cessation (Figure 6). From 2017, the trend in the increase in the number of vessels of the other two segments (DTS 06–12 and DTS 24–40) should not be read as an increase in the number of licenses for trawling; rather, it should be viewed as an increase in the use of trawl nets as the predominant type of fishing gear^12^.

**Figure 7:**
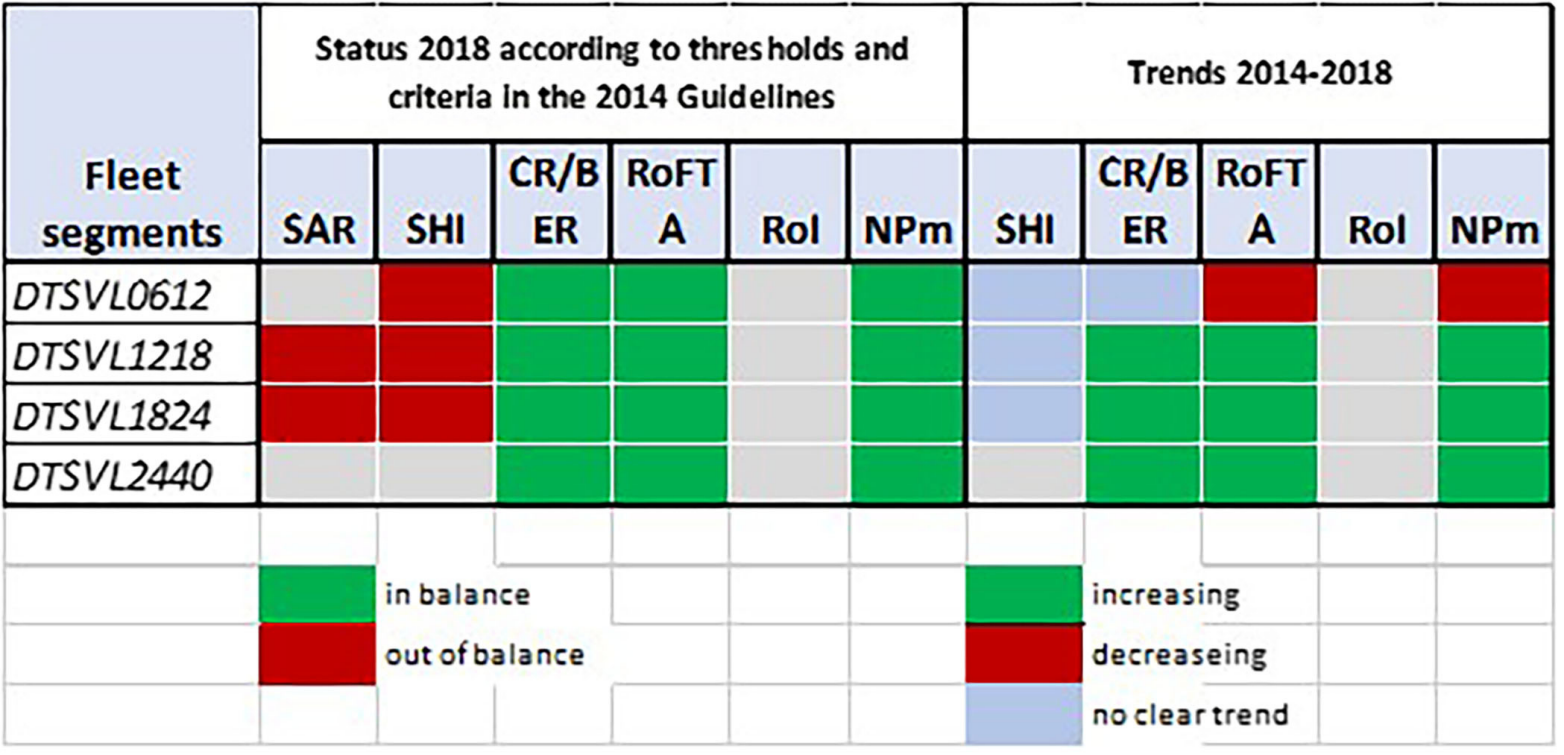
Balance indicators for the Italian demersal trawl fleet in 2018 and the trend for the 2014–2018 period. Elaboration on data from STECF (20-10).

To properly evaluate the use of the permanent cessation measure, the balance indicators considered by the STECF (20-11) to provide an assessment about the existence of balance/unbalance between a fleet's capacity and fishing opportunities were considered. Regarding the biological risk dimension, in 2018, the Italian demersal trawl fleet appears to have been in the unbalance category (Figure 7).

The Stocks-At-Risk (SAR) indicator measures the number of fish stocks that are exploited by the fleet and that are assessed as being at high biological risk based on different criteria, including if the stock is below the spawning stock biomass reference point, which is identified as the stock size below which a population has a high likelihood of being impaired.

The sustainable harvest indicator (SHI) reflects the extent to which a fleet segment depends on overfished stocks. Overfished means that a stock is fished at a fishing mortality rate above the fishing mortality rate corresponding to the maximum sustainable yield (F_MSY_). Exceeding the upper limit of the F_MSY_ range is interpreted as overfishing.

**Figure 8:**
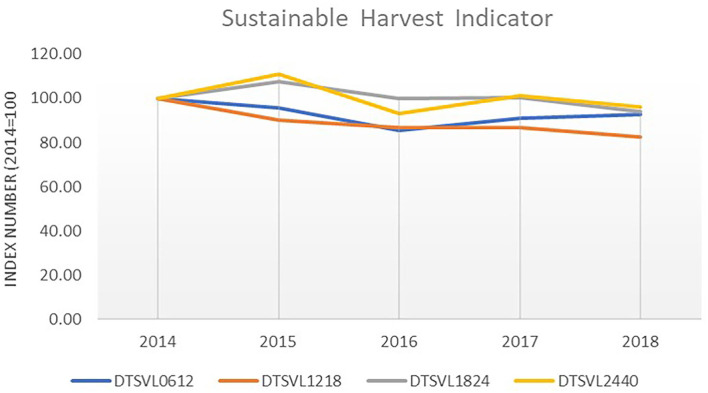
SHI trend for the Italian demersal trawl fleet for the 2014–2018 period. Elaboration on data from STECF (20-10).

Although the Italian demersal trawl fleet was not in the balance category for 2018, a decreasing trend emerged for SHI^13^ (meaning progress has been made toward achieving balance) for the 2012–2018 period for all the trawl fleet segments (−9%), specifically for boats included in the length class of 12–18 m (−18%), one of the two most funded segments under Art. 34 (Figure 8).

However, from a socio-economic perspective, balance is also suggested by the indicators regarding the current revenue as a proportion of the break-even revenue (CR/BER), the return on fixed tangible assets (RoFTA), and the net profit margin (NPm) which is a percentage of revenue that a fleet segment retains as profit and measures the amount of surplus generated per unit of production.

Nevertheless, it is interesting to report the trend of the economic dependency indicator (EDI), highlighting the fleet segments that more heavily rely on overfished stocks. The EDI indicator may help MMs prioritize their actions according to how financially dependent the different fleet segments are on overfished stocks.

**Figure 9:**
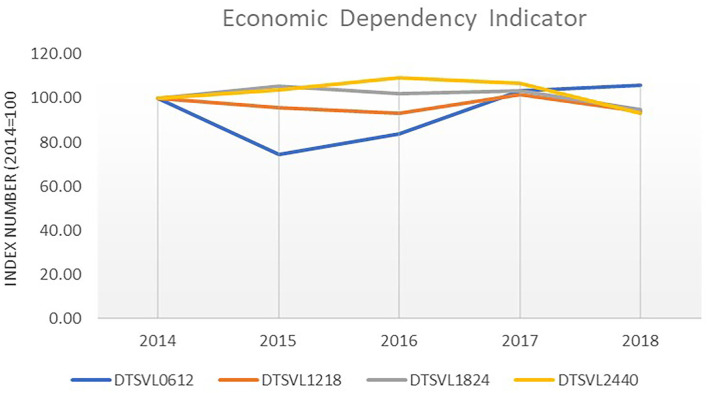
EDI trend for the Italian demersal trawl fleet for the 2014–2018 period. Elaboration on data from STECF (20-10).

Figure 9 reports the EDI trend for the Italian demersal trawlers for the 2014–2018 period highlighting a small decrease (-6%) for demersal trawlers longer than 12 m and an increasing trend only for trawlers in the smallest length class.

#### Fuel efficiency

**Figure 10:**
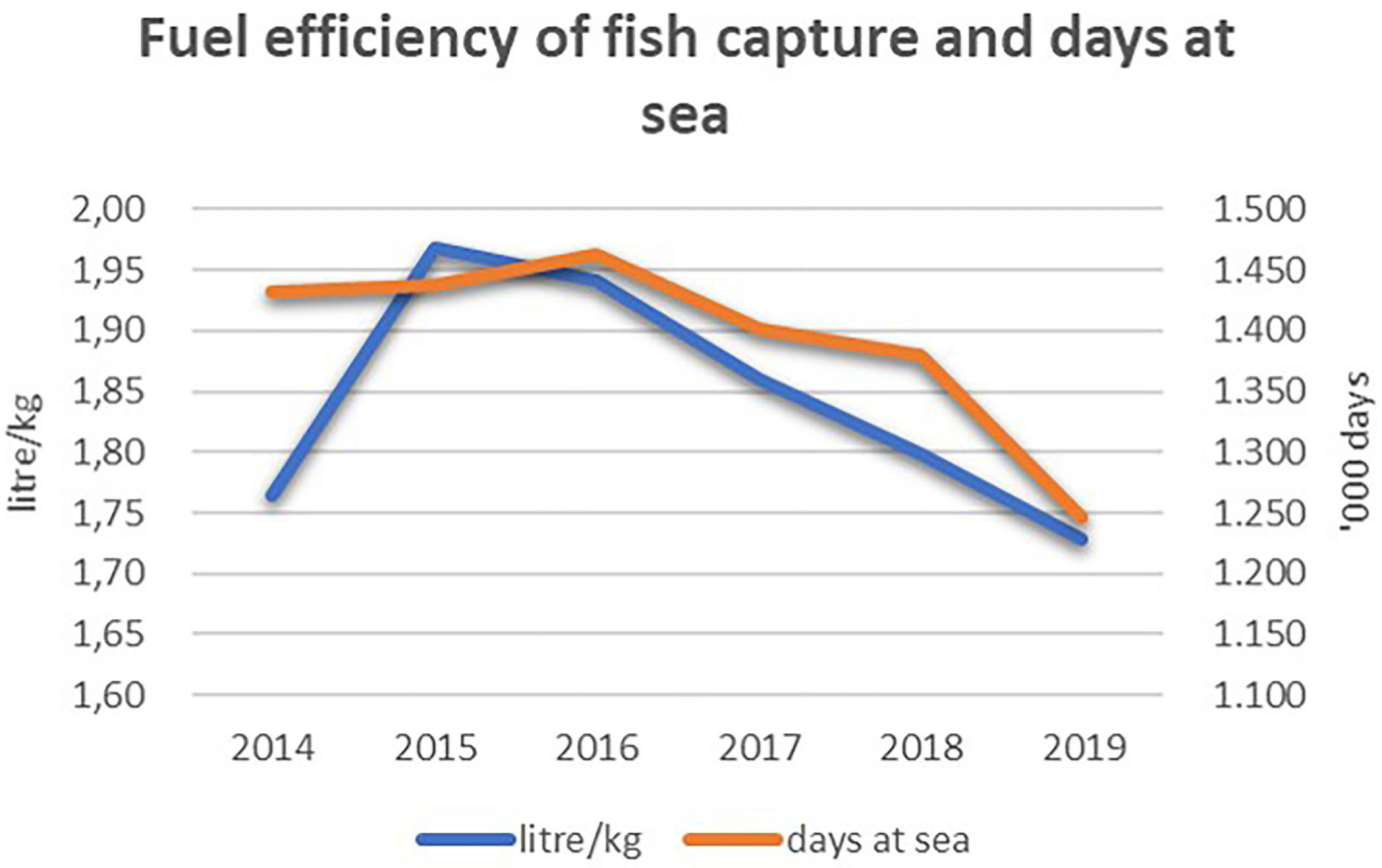
Fuel efficiency ratio and number of days at sea of the Italian fishing fleet, 2014–2019. Elaboration on data from STECF (20-10).

For the measure under Art. 41, it was not possible to conduct an in-depth analysis, i.e., at the fleet segment level, as the vessel identification number was only reported for six of the 84 operations specifically referred to in the measure under Art. 41.2 (engine replacement). Figure 10 shows the results of an alternative analysis of the performance of the fuel efficiency of the entire Italian fishing fleet based on the ratio between energy consumption and fish landed (blue trend on the left side of Figure 10).

The decreasing trend in the use of fuel per kilogram of landings implies a reduction in carbon emissions, indicating a positive effect on the environment. Nevertheless, based on the overall amount of funds allocated to this measure, equal to 0.52% of the overall EMFF allocated funds in Italy, let us assume that the positive change in the fuel efficiency is most likely due to the decrease in the number of the days at sea from 2016 (orange trend on the right side of Figure 10).

### Intensity metrics analysis

In a second step, the analysis was based on the use of intensity metrics. This allowed for standardizing the subsidization, as was done on a wider level by Skerritt and Sumaila (2021). Harmful fishery subsidies can cause substantial damage to fish stocks when they, directly or indirectly, artificially inflate profitability. Indeed, profitability is a key driver of overcapacity and overfishing. Very often, a significant amount of attention is paid to the provision of large sums of subsidies to specific fishing fleets or countries, but one key issue is the need to provide a measure of the potential scale and relative impact of fishery subsidies. According to Skerritt and Sumaila (2021), p. 2: “Given that the impact of an injection of public funds in this manner is primarily an economic one with subsequent deleterious ecological and social consequences, clearly the harm caused by subsidies must, to some extent, be relative to the economic scale of the fishery.”

Considering the absolute value of the subsidies alone, not understanding the context within which they are provided results in ignoring the “pervasive impacts of subsidies unrelated to their scale, such as their inequitable distribution” (Skerritt and Sumaila, 2021, p. 1). Using recently available data and various scaling analyses, we developed a series of different intensity subsidy metrics to broaden our understanding of the distribution of EMFF among EU MSs.

The intensity metrics were developed using the following steps:

1. Elaboration of the EMFF 2014–2020 allocated funds by type (beneficial, ambiguous, and capacity enhancing/harmful) and by EU Mediterranean countries (based on the data collection and categorization).
2. Extraction of data on the economic scale of the fisheries for each selected country from:
  a. The dataset of the Annual Economic Report of the EU fishing fleets containing data on the dimensions of the fleets (number of vessels), available at https://stecf.jrc.ec.europa.eu/dd/fleet;
  b. The dataset of the Fishery Dependent Information (FDI), available at https://stecf.jrc.ec.europa.eu/dd/fdi, for data on the dimensions of the production (landings) by country, volume, and value.
3. Generation of simple subsidy intensity metrics given by the ratio between the EMFF funds (bullet 1) and data on the economic scale (bullet 2) per each country;
4. Generation of the standardized subsidy intensity metrics calculated as a proportion of the total, to easily compare and present all of metrics in the same graph.

Data on the overall amount of allocated funds (total public expenditure and EMFF contribution) for the 2014–2020 programming period by type of subsidies (beneficial, ambiguous, and capacity enhancing/harmful) and by EU countries (bullet 1) are reported in Table 5. Only funds allocated under UP1 (promoting environmentally sustainable, resource-efficient, innovative, competitive, and knowledge-based fisheries) and primarily associated with fishing boats were considered in the present analysis, considering that the intensity metrics are based on the dimensions of the EU Mediterranean fishing fleets.

**Table 5:** Number of financed operations, total public expenditures and EMFF contributions by MSs and subsidy category.

		**Ambiguous**	**Beneficial**	**Capacity enhancing**	**Total**
Croatia	No. financed operations	1.561	147	30	1.738
	Total public expenditure (€)	37.281.546	2.814.027	813.528	40.909.100
	EMFF contribution (€)	18.638.641	1.163.400	559.637	20.361.678
France	No. financed operations	88	11	12	111
	Total public expenditure	810.800	2.405.834	811.042	4.027.675
	EMFF contribution	535.546	993.969	247.639	1.777.154
Greece	No. financed operations	664	190	151	1.005
	Total public expenditure	40.676.188	18.128.521	63.995.941	122.800.650
	EMFF contribution	40.676.188	16.930.702	63.018.789	120.625.679
Italy	No. financed operations	6.632	475	151	7.258
	Total public expenditure	82.852.824	49.128.727	42.759.255	174.740.806
	EMFF contribution	82.852.824	49.128.727	42.759.255	174.740.806
Malta	No. financed operations	26	2	9	37
	Total public expenditure	100.621	700.000	10.279.610	11.080.231
	EMFF contribution	50.311	525.000	7.595.207	8.170.517
Spain	No. financed operations	1.129	612	117	1.858
	Total public expenditure	16.314.614	22.110.996	7.238.805	45.664.415
	EMFF contribution	8.157.306	15.925.111	4.420.527	28.502.944

Elaboration on data from the EMFF list of operations.

Data to adjust the subsidization according to the economic scale of the fishery sector for each country (bullet 2), i.e., the fleet (number of vessels) and production size (volume and value of landings), are reported in Table 6.

**Table 6:** Average number of active vessels, volume and value of the landings by MSs for 2017–2019.

**EU**	**Number of**	**Total live weight**	**Total value of**
**MS**	**vessels**	**landed (tons)**	**landings (euro)**
Croatia	7.970	67.362	55.878.158
France	1.464	16.208	116.523.878
Greece	14.431	47.001	286.004.865
Italy	12.140	180.594	904.011.953
Malta	928	1.433	9.568.746
Spain	2.487	76.971	306.667.852

Elaboration on data from the Annual Economic Report (STECF, 2020-06) and FDI (STECF, 21-08). 1) the average volume and value of the landings for Greece is estimated for 2018–2019; 2) the landings for Spain and France only refer to fleets active in the Mediterranean Sea as available in (STECF, 20-10).

**Figure 11:**
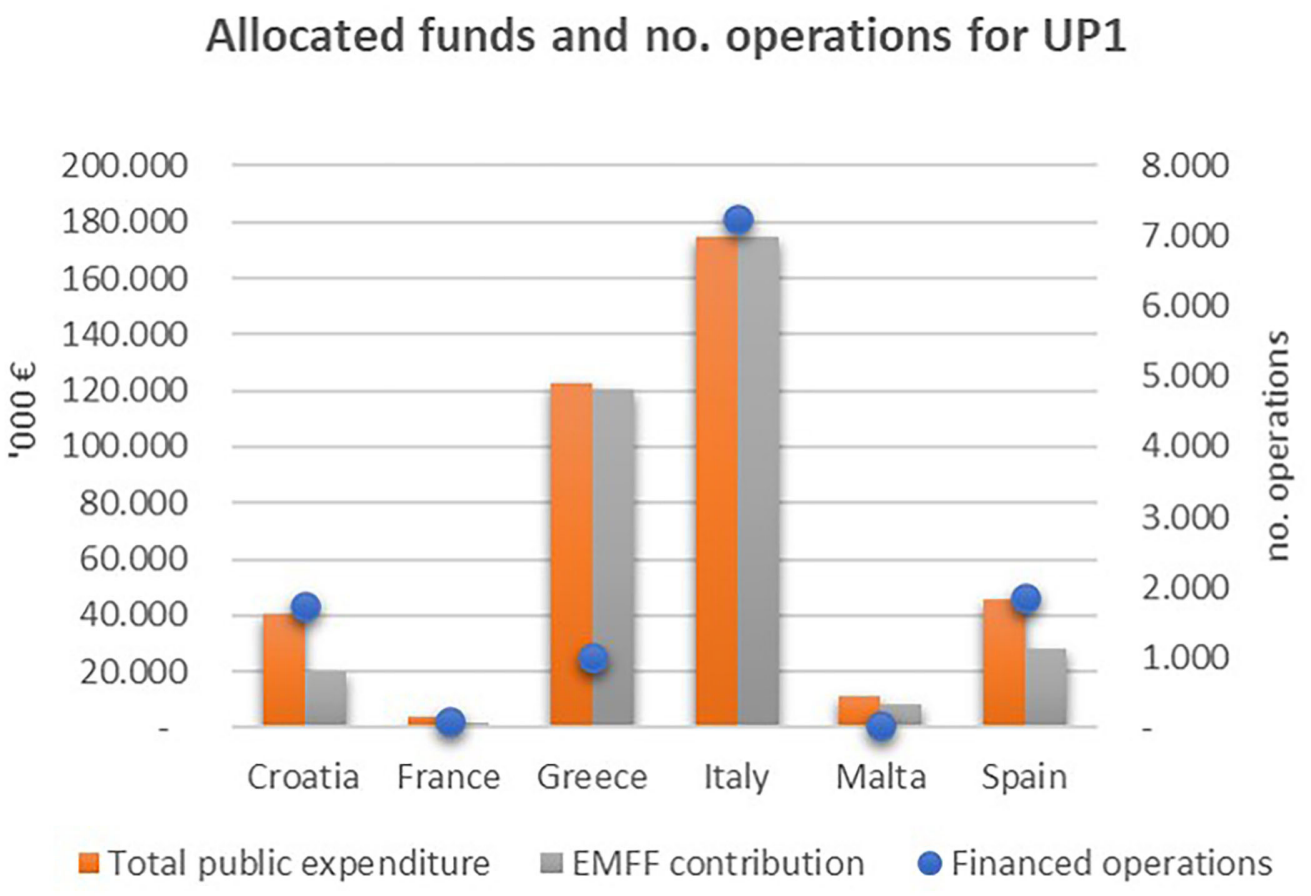
Public expenditure, public contribution and number of operations allocated to the UP1 – (promoting environmentally sustainable, resource-efficient, innovative, competitive and knowledge-based fisheries) by MSs. Elaboration on data from the EMFF List of Operations.

As highlighted in Figure 11, when simply considering the overall amount of funds allocated under EMFF and the number of operations under UP1 (promoting environmentally sustainable fisheries), Italy is the top subsidized country, receiving around €175 million of the EFF allocated funds and more than 7,000 financed projects. Greece follows in terms of amount of money allocated, even though that country's fishing fleet is larger than Italy's. Spain and Croatia are, respectively, second and third in terms of the number of financed projects, even though the contribution of EMFF to the total public expenditure is lower than it is in Italy and Greece.

**Figure 12:**
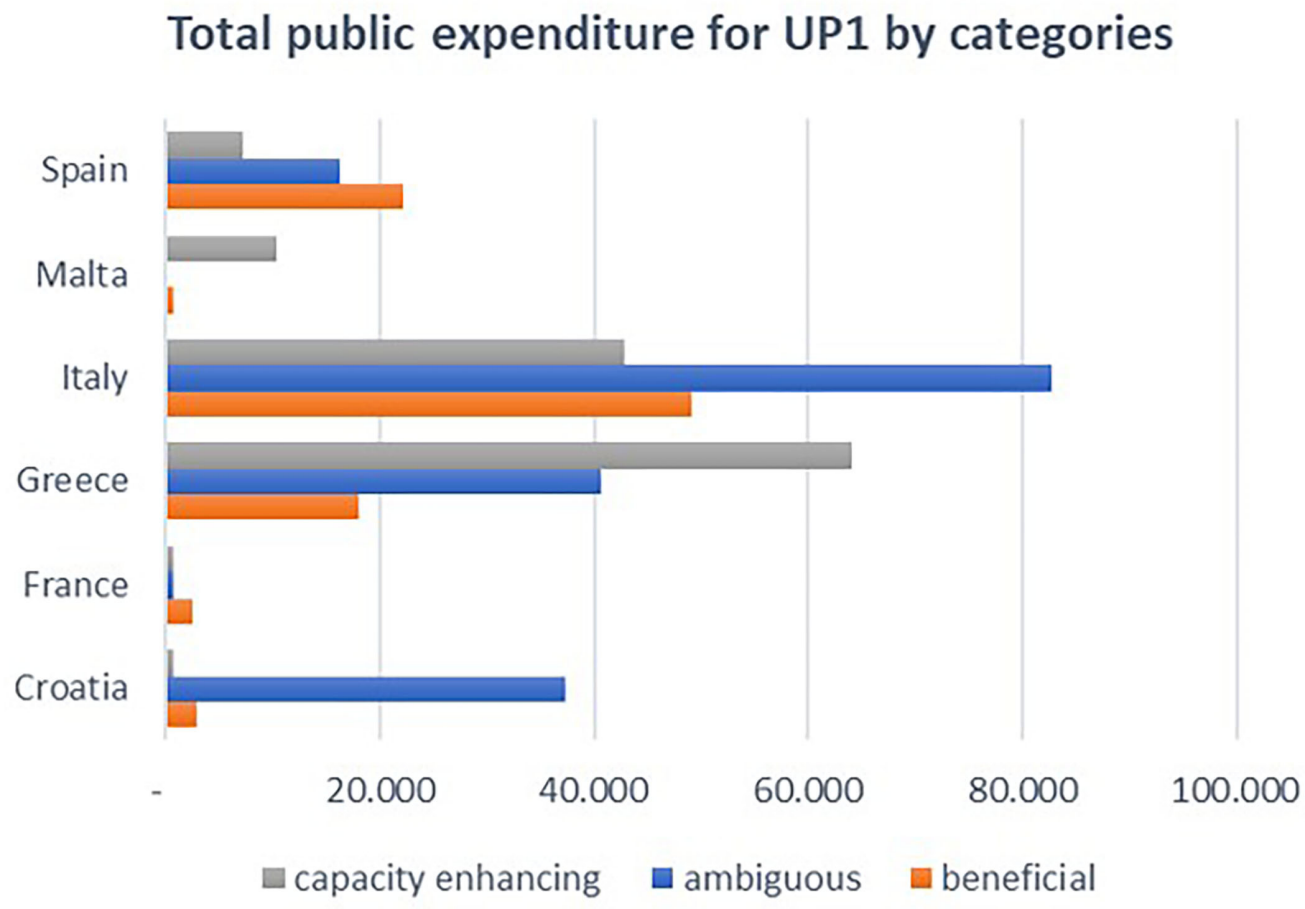
Total public expenditures of UP1 measures by category and MSs. Elaboration on data from the EMFF List of Operations.

The analysis of the total funds by categories (Figure 12) shows that, while beneficial subsidies are the largest category for Spain and France, in Italy and Croatia, ambiguous subsidies are the largest category (for permanent cessation of fishing activity in Italy and temporary cessation in Croatia). Capacity-enhancing subsidies are the largest category in Greece and Malta (funds allocated for fishing ports, landing sites, auction halls, and shelter-related projects).

**Figure 13:**
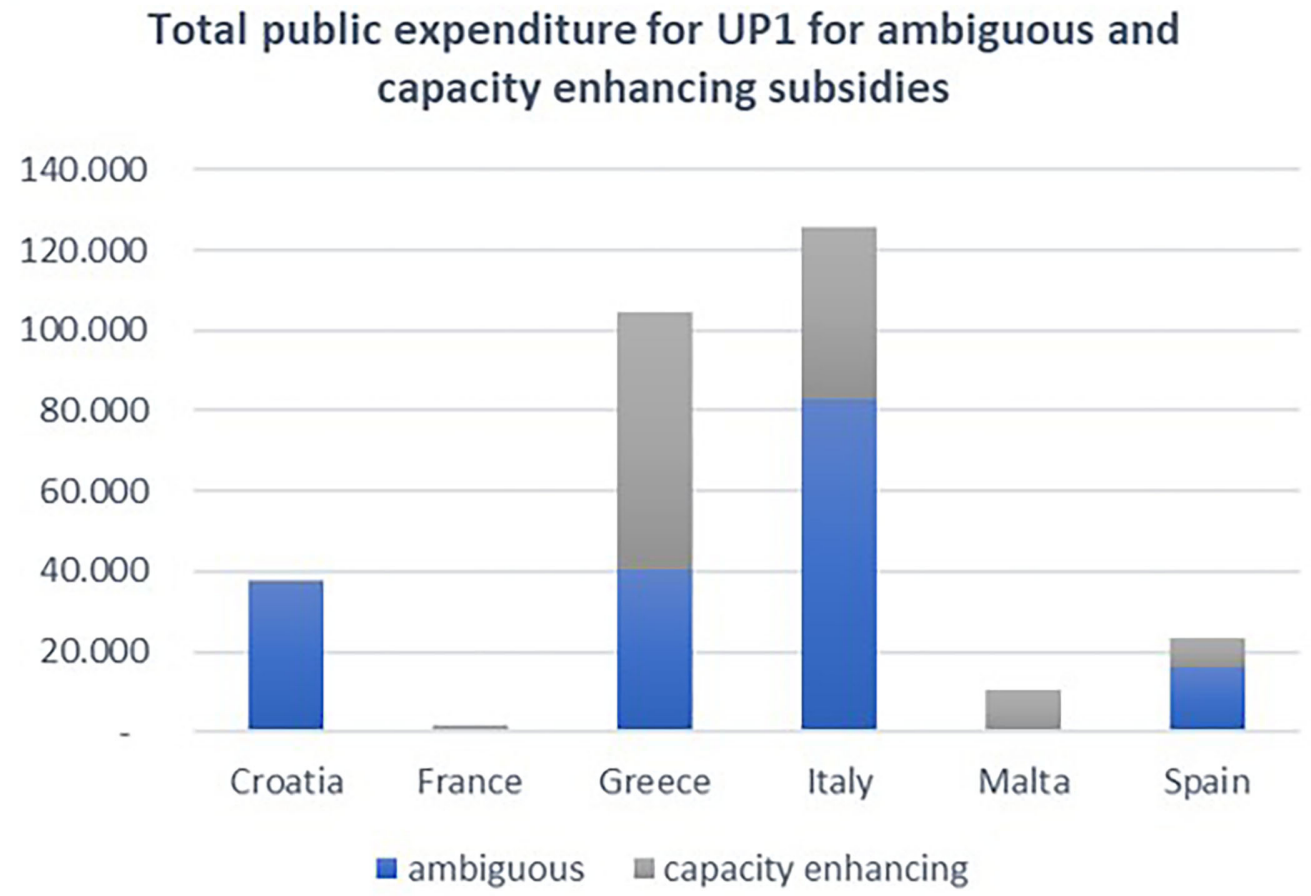
Total public expenditures for the UP1 ambiguous and capacity-enhancing measures by EU MSs. Elaboration on data from the EMFF List of Operations.

Italy is the top subsidized country under UP1 if only considering the funds allocated under the full or potential harmful subsidies, i.e., ambiguous plus capacity enhancing (Figure 13).

**Figure 14:**
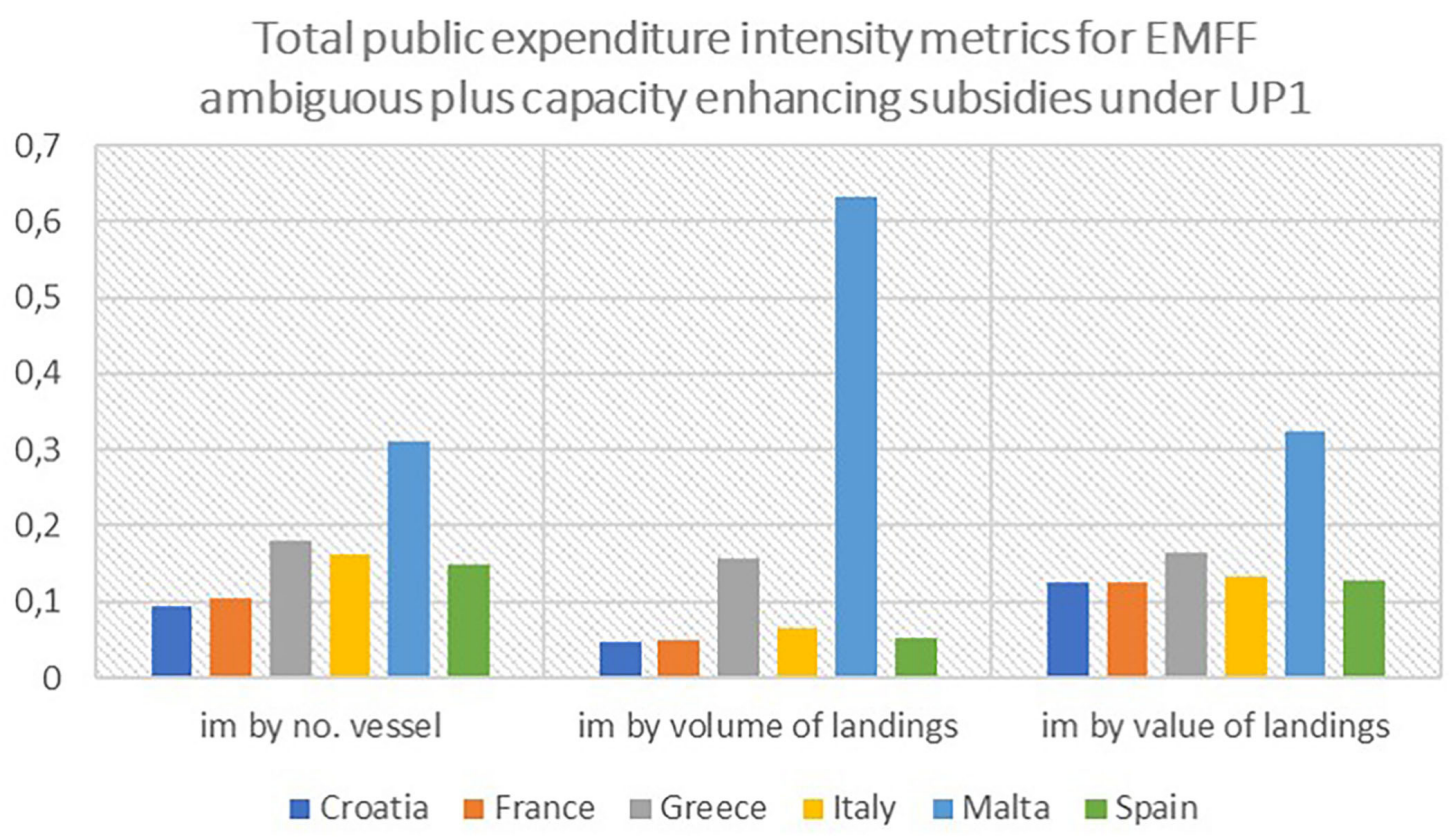
Total public expenditure intensity metrics for ambiguous and capacity-enhancing subsidies by MSs. Source: elaboration on data from EMFF lists of operations.

Although EMFF's contribution could be underestimated for some countries (taking into account differences in the implementation rate for EMFF among MSs), the use of the subsidies' intensity metrics allowed for an in-depth analysis, resulting in completely different findings. Figure 14 shows that countries can be ranked as a top receiver of subsidized funds, depending on the metrics that are used. When the selected intensity metrics were used, the Maltese fishing fleet, as the fifth ranked in terms of absolute value of allocated funds, becomes the top subsidized fleet in the Mediterranean region, followed by Greece. The situation changes even more when the intensity metrics are calculated based on the volume and value of the landings; in that scenario, the amount of subsidized funding allocated to Malta is even than the funding received by Greece and other countries. The Italian fishing fleet appears to only be ranked the third highest subsidized fleet, and very close, in proportional terms, to Spain, France, and Croatia.

### Qualitative analysis of the EU subsidy policy in the fisheries

The quantitative assessment of the EMFF was complemented with stakeholder interviews to provide additional information and qualitative perspectives on the EU subsidy policy. Interviews were held with six stakeholders^14^ who have received beneficial subsidies and have an interest in the environmental sustainability and fishery sector, for a total of four case studies. The interviews were based on a template containing semi-structured questions to understand the context within which the EMFF operates, to obtain the stakeholders' perceptions and use of the EMFF in relation to key issues facing the marine ecosystems and to identify best practices.

All the stakeholders highlighted that the active participation of fishers is a crucial factor for successful projects. The involvement of local operators is based on mutual trust with the project promoters, and it is gained over time based on mutual benefit. For instance, establishment of the marine protected area of Punta Campanella in Southern Italy represents an additional source of income for local fishers who received financial incentives for testing new, less impactful fishing gears or from the creation of additional no fishing zones. The Sepoline project, which aimed to prevent the destruction of cuttlefish eggs in the North Adriatic Sea, was successful because it used new types of egg collectors mounted on the pots to solve the resource depletion issue experienced by local fishers. The capacity to define, prepare, and develop cooperation projects and establish partnerships among operators, scientists, policymakers, and civil society is the strength of the Italian and Spanish FLAGs. These organizations have extensive experience in promoting competition and innovation in small-sized enterprises, in valuing environmental and cultural assets in the area and developing innovative tourism products. These initiatives have increased the environmental awareness of local operators and fishers.

Expressed mistrust in the regional authorities was similar in all the investigated case studies. Regional administrations are accused of being inefficient and having a lack of strategic planning. Some of the respondents stated that the regional administrations are mostly interested in achieving the expenditure targets without proper concern for both the evaluation phase and future progress of the projects. The unclear sharing of responsibilities among different levels of government further complicated the procedures, imposing extra requirements and administrative burdens (e.g., duplication of financial audits). Consequently, the double-checking system by national and regional authorities significantly slowed implementation of the payment procedures. The waiting times for payments increased while the reporting obligations become more and more stringent.

The interviewees also emphasized the quantity of the bureaucratic constraints, e.g., of the high number and types of authorisations requested for undertaking some projects, such as those concerning fishing tourism activities. The stakeholders also highlighted the difficulty in finding a regulatory and scientific reference (accepted by the regional administration) for the remuneration of the activities carried out by fishers (e.g., marine litter projects).

Finally, the ability to continue the activity after exhausting the EMFF resources is another important drawback. Even when projects proved to be successful, continuation is not always guaranteed because the additional costs for investments in new technologies and fishing gears must be exclusively borne by fishers. The management authority should implement the necessary operations and infrastructures to ensure the continuation of worthwhile innovative activities. The new programme could capitalize on the positive results obtained in the previous period by completing activities that are financed under the EMFF, but require additional financial support.

## Discussion and final conclusions

Constructing an inventory of EMMF funds in the Mediterranean region allowed us to conclude that the environmental dimension of CFP accounts for a small share of the EMMF budget implemented in the Mediterranean. Between 2014 and 2020, most of the EU financial support to the fishery sector was used to improve economic growth by increasing production and reducing overcapacity. UP1 (promoting environmentally sustainable, resource-efficient, innovative, competitive, and knowledge-based fisheries) had the largest number of operations reported by the six MSs (32% of the total) and EMFF measures specifically designated to fishing vessels were allocated almost entirely to the ambiguous category (temporary cessation of the fishing activities [EMFF Art.33 and to withdrawal [EMFF Art. 34]). The measure concerning energy efficiency and mitigation of climate change under EMFF Art. 41 has a very low rate of implementation.

According to information collected from the operation lists of five countries (Italy, France, Spain, Greece, and Malta) for which data on individual vessels were available, trawlers longer than 12 m, accounting for 14% of the total registered fleet in these five countries, received 75% of the total approved projects and 71% (€100 million) of the total budget granted to fishing vessels. Vessels using static gear less than 12 meters in length, classified as an SSCF and representing three-quarters of the total Mediterranean fleet, received 3% of the total EMFF funding specifically designed for fishing vessels (under UP1), both in terms of the number of operations and the allocated funding amount. Although this total figure can be underestimated by considering that most small-scale vessels are the members of cooperatives and receive funding through these organizations, there is a clear imbalance in the use of public funding between fleets with small-scale vessels and those with large-scale vessels. In fact, EMFF financial support to vessels not using trawling gear, specifically funding of small-scale vessels, mainly contributed to the improvement and diversification of activities, the enhancement of the competitiveness, and the viability of the fisheries. Funding provided to larger trawl vessels was limited to compensating for the exit from the sector or for the temporary cessation of fishing activities.

An in-depth evaluation of the fishery subsidy policy requires the identification of productivity, biological, and capacity indicators to measure the level of subsidization by fleet segments. This enables one to verify the potential harm of the subsidies and determine if the subsidies impact the biological targets established in the operational programmes for scrapping measures (EMFF Art. 34.1) and in the management plans for the temporary cessation (EMFF Art. 33.1). However, in most cases, information provided on the operations lists is misreported or missing, as in the case of the CFR number for most measures linked to fishing vessels. In particular, the analysis of the available information highlighted that, for specific measures, such as permanent cessation of fishing activities and engine replacement, there is not enough data to conduct a specific quantitative analysis, so it is not possible to reach clear conclusions. However, due to the limited amount of money allocated on this measure, we concluded that the weak signs of improvement in terms of fuel efficiency are more likely due to other concomitant causes (reduced fishing days). Regarding permanent cessation (Art. 34), from the analysis done on the fleets that received the most subsidies in Italy (demersal trawlers ranging in length between 12 and 24 m), it was found that, to a certain extent, the measure contributes to the achievement of the envisaged objectives.

The classification of the measures according to the EMFF articles is another critical issue that makes the information provided by MSs not always transparent and consistent. Although some specific projects are classified under different subparagraphs of the same EMFF article, in the operations lists, most projects are only provided by the main articles aiming to pursuit different policy objectives. Consequently, based on the information reported by some of the MSs, it was not possible to assess the level of implementation of measures, such as those concerning NATURA 2000 sites (EMFF Art. 40.1, landing obligations (Art. 38a, Art. 38b, Art. 42.b, and Art. 43.2) and the engine replacement of fishing vessels (Art. 41.2).

The analysis has also demonstrated that the metrics used to evaluate the entity of the subsidization affects the results of the assessment. The estimations of most of the subsidized fleets change if the EMFF contribution is based on absolute monetary values or on weighted metrics estimated in relation to the number of vessels, the volume, and the value of the landings. Recent studies in the literature have highlighted that, very often, a significant amount of attention is paid to the provision of large sums of subsidies to specific fishing fleets or countries, but the key issue of providing a measure of the potential scale and relative impact of fishery subsidies remains unreported. Considering the absolute value of subsidies alone, not understanding the context within which they are provided may result in ignoring the “pervasive impacts of subsidies unrelated to their scale, such as their inequitable distribution” (Skerritt and Sumaila, 2021, p.1). Using recently available data and various scaling analyses, a series of different subsidy metrics was developed to broaden the understanding of the distribution of EMFF among Mediterranean MSs. The main finding of this analysis is that countries could be top beneficiaries depending on the metrics that were used. When considering the absolute value of subsidization, focusing exclusively on ambiguous and capacity-enhancing subsidies under UP1 (promoting environmentally sustainable fisheries), Italy is the main subsidized country. This is understandable if one considers that Italy and Greece have the largest fishing fleets of the six studied countries. Using the subsidy intensity metrics based on the number of vessels and on the volume and value of landings, the situation changes completely: Malta becomes the top subsidized country while Italy falls to third place. In the future work, this empirical analysis could be easily extended at the fleet segment level to evaluate the relative subsidization for large-scale vessels and small-scale vessels. Furthermore, data could be updated to the last year of the programming period, 2022, to consider that, at 2020 most large MSs, such as Italy and Greece, did not spend all the funds in their total allocated budget.

The asymmetric implementation of the measures depends on a combination of different factors, many of which have been raised in previous studies and reports evaluating the fishery funds of previous programming periods (MRAG, 2013; Ballesteros et al., 2018). Bureaucratic hurdles, low co-financing rates, and ambiguous eligibility criteria have reduced the attractiveness for most innovative and longer-term measures and have hampered the participation of the private sector. Limited budgets and difficulty accessing financial instruments have penalized most of the projects that focus on innovation and diversification, which require private co-financing. Excessively detailed and ambiguous eligibility criteria affected the applicability of some of the measures, such as those concerning young fisher start-ups and training. In some countries, the authorities in charge of managing the operational programmes further complicate the procedures by imposing extra requirements and administrative burden. The lack of coordination between EU legislation and national laws makes the proper use of the funds even more complex. Moreover, in larger countries, such as Italy and Spain, the use of the funds is divided among various administrators and is decentralized to the regional level.

In the interviews, some of the Spanish and Italian stakeholders involved in successful environmental projects provided interesting insights into the results of the analysis. In all the interviews, the critical role played by regional authorities emerged, which further contributes to the dispersion of the responsibilities among people in all levels of government tasked with implementing the EMFF programme. This also contributed to the slowdown of projects. The interviews highlighted the difficulties the stakeholders encountered with national and regional administrations in terms of the consultation process and strategic planning. The Spanish case study highlighted how the increasing decentralized management at the regional level has had a negative impact on the activity of a local FLAG with a long and successful tradition in the management of structural funds. Moreover, the low percentage of EMMF funding received by SSCFs can be explained by the complexity and inefficiency of the procedures used to access financial support that discourage moving forward in terms of sustainability, innovation, and the competitiveness of small-scale fisheries.

The possibility of continuing the activities after the completion of the projects and exhausting of the European financial resources are other drawbacks stressed by the interviewees. Once a project is completed, its financing ends and there is the risk that even the most valuable activities cease. The new European Maritime, Fisheries, and Aquaculture Fund (EMFAF) 2021–2027 should capitalize on the positive results obtained in the previous programming period and amend the eligibility criteria for the extension of projects and contracts that proved to be successful and/or need additional financial resources to continue.

Dealing with the problems strongly perceived by operators is the main requirement for the involvement of stakeholders and, thus, for the success of future programming. Therefore, another important factor that impacts the success of the financed projects is related to the capacity to establish partnerships between all the actors (operators, scientists, policymakers, and civil society) that can combine growth and sustainability, while respecting the territorial specificities (De Boni et al., 2018). The construction of functional partnerships between research and operators is also an essential prerequisite to stimulate investments in new “green” technologies or activities, which often need a significant level of financial resources and the engagement of and acceptance by all operators. From this perspective, more aid and encouragement should be given to FLAGs and other local groups to promote cooperation and increase local competences, and to vocational schools, allowing for generational turnover but also alternative job opportunities.

## Data availability statement

The datasets presented in this study can be found in online repositories. The names of the repository/repositories and accession number(s) can be found in the article/Supplementary material.

## Author contributions

MGa or MGo developed the database, the quantitative assessment of funds allocation by measures and fleets, and wrote the article. LM developed the analysis on the impact of measures 34 and 41. All authors contributed to the design and implementation of the research, to the analysis of the results, to the writing of the manuscript, and approved the submitted version.

## Funding

This research was supported by WWF Mediterranean Initiative - MAVA Co-managed No-Take Zones/MPAs Project.

## Conflict of interest

The authors declare that the research was conducted in the absence of any commercial or financial relationships that could be construed as a potential conflict of interest.

## Publisher's note

All claims expressed in this article are solely those of the authors and do not necessarily represent those of their affiliated organizations, or those of the publisher, the editors and the reviewers. Any product that may be evaluated in this article, or claim that may be made by its manufacturer, is not guaranteed or endorsed by the publisher.

## Footnotes

^1^Slovenia and Cyprus were excluded from the analysis because information on EMFF projects was not accessible due to privacy issues (given the limited the number of beneficiaries) or not provided in English.

^2^The website portal Available online at: https://euribarstvo.hr/operativni-program-za-pomorstvo-i-ribarstvo-rh-za-programsko-razdoblje-2014-2020/ provided the list of beneficiaries and operations updated to 31st August 2019.

^3^The website portal Available online at: https://www.europe-en-france.gouv.fr/fr/fonds-europeens/fonds-europeen-pour-les-affaires-maritimes-et-la-peche-FEAMP provided the list of beneficiaries and operations updated to 7 December 2020. Only measures related to beneficiaries located in the Mediterranean region (Corsica and the coast of the Gulf of Leon) were considered. To estimate the amount of the financial execution only projects, the category soldè and the sub-category montant total engagé payé were selected.

^4^The website portal Available online at: https://anaptyxi.gov.gr/en-us/PROJECTS-GRANTS provides information on, and access to, the operational programme in Greece. Data are updated to June 2020 and are available at NUTS2 level.

^5^The website portal Available online at: https://opencoesione.gov.it/it/programmi/2014IT14MFOP001/ provided information on the operational programme updated to December 2020 and are available at NUTS2 level.

^6^The link to the EMFF database webpage (https://emff.gov.mt/emff1420/) was updated to 2nd December 2020.

^7^The website portal Available online at: https://www.mapa.gob.es/es/pesca/temas/fondos-europeos/transparencia-femp-30-06-2020-v14_tcm30-526636.pdf provided the list of beneficiaries and operations updated to June 2020. Only the measures in Mediterranean regions (Valencian Community, Andalucía, Cataluña, Balearic Islands, Región de Murcia, Ciudad Autónoma de Ceuta) and provided for by national administration bodies were considered.

^8^Commission Regulation (EC) No., 26/2004 of 30 December 2003 on the Community Fishing Fleet Register.

^9^According to Table 2 (Fishing activity-metier) of Commission Decision (EU) 2016/1251.

^10^According to the DCF definitions, SSCF includes all vessels under 12 metres using static gears: ‘drift and/or fixed netters', ‘pots and/or traps', ‘hooks', ‘passive gears only', ‘other passive gears', ‘polyvalent passive gears only', ‘active and passive gears'. Large-scale fleet (LSF) includes all vessels over 12 metres using static gears and all vessels using towed gears operating predominately in EU waters. These include: ‘dredgers', ‘demersal trawlers and/or demersal seiners', ‘other active gears', ‘polyvalent active gears only', ‘purse seiners', ‘beam trawlers', ‘pelagic trawlers'.

^11^Regulation (EU) 2020/460 of the European Parliament and of the Council of 30 March 2020 amending Regulations (EU) No 1301/2013, (EU) No 1303/2013 and (EU) No 508/2014 regarding specific measures to mobilise investments in the healthcare systems of Member States and in other sectors of their economies in response to the COVID-19 outbreak (Coronavirus Response Investment Initiative).

^12^DTS is the acronym for demersal trawlers as foreseen by the EU classification of fishing fleet segments available at https://datacollection.jrc.ec.europa.eu/wordef/fleet-segment-dcf.

^13^The trend is not evidenced for SAR because there is no continuity on the Med stocks assessed each year.

^14^The director of the marine protected area of Punta Campanella; two researchers involved in the Sepoline project; the director of the Costa Emilia Romagna FLAG and the director of the Litoral Cádiz Estrecho” FLAG.

## Abbreviations

BER: Break-Even Revenue
CFP: Common Fisheries Policy
CLLD: Community Local Led Development
CFR: Community Fleet Register
CR: Current Revenue
RoFTA: Return on Fixed Tangible Assets
DCF: Data Collection Framework
DTS: Demersal Trawlers
EDI: Economic Dependency Indicator
EMFAF, European Maritime, Fisheries: and Aquaculture Fund
EMFF: European Maritime and Fisheries Fund
EU: European Union
FAME: European Union Fisheries and Aquaculture Monitoring & Evaluation
FDI: Fishery Dependent Information
FLAG: Fishery Local Action Group
LOA: Length Overall of a vessel's hull measured in meters
LSF: Large-scale fleet
MPA: Marine Protected Area
MS: Member State
NPm: Net Profit margin
NUTS: EU Nomenclature of Territorial Units for Statistics
SAR: Stocks-At-Risk indicator
SHI: Sustainable Harvest Indicator
SSCF: Small-scale Coastal Fleet
UP: Union Priority.
